# An application of the theory of planned behavior to self-care in patients with hypertension

**DOI:** 10.1186/s12889-020-09385-y

**Published:** 2020-08-26

**Authors:** Gholamreza Pourmand, Leila Doshmangir, Ayat Ahmadi, Mohammad Noori, Atiyeh Rezaeifar, Rahil Mashhadi, Rezvan Aziminia, Amirhossein Pourmand, Vladimir S. Gordeev

**Affiliations:** 1 Urology Research Center, Tehran University of Medical Sciences, Tehran, Iran; 2 Department of Health Policy and Management, Tabriz Health Services Management Research Center, School of Management and Medical Informatics, Tabriz University of Medical Sciences, Tabriz, Iran; 3 Iranian Center of Excellence in Health Management, School of Management and Medical Informatics, Tabriz University of Medical Sciences, Tabriz, Iran; 4 Social Determinants of Health Research Center, Tabriz University of Medical Sciences, Tabriz, Iran; 5 Knowledge Utilization Research Center, Tehran University of Medical Sciences, Tehran, Iran; 6 Sina Hospital, Tehran University of Medical Sciences, Tehran, Iran; 7 Institute of Population Health Sciences, Queen Mary University of London, London, UK; 8 Department of Infectious Disease Epidemiology, London School of Hygiene & Tropical Medicine, London, UK

**Keywords:** Theory of planned behavior, Self-care, Hypertension, Lifestyle

## Abstract

**Background:**

Self-care behaviors and positive changes in lifestyle are essential for successful hypertension control. We used a behavioral model based on the theory of planned behavior to assess which factors influence self-care behaviors for controlling hypertension.

**Methods:**

In this cross-sectional study, five hundred patients with at leastaone-year history of diagnosed hypertension participated in this study. The data collection tool was designed based on the theory of planned behavior. Structural equation modeling was used to estimate the main parameters.

**Results:**

For self-care behaviors, ninety-six (19.2%) and forty-five (9.1%) participants had good knowledge and acceptable behavior(≥8 out of 10 points). Having perceived behavioral control regarding quitting smoking and alcohol intake was associated with the patient’s intention and behavior [b:1.283 ± .095 and b:1.59 ± .014 (*p* < .001)]. Having perceived behavioral control over the other self-care behaviors had a positive effect on the intention in female patients [b: .885 ± .442 (*p* = .045)]. Subjective norms had a positive effect on behavioral intention in younger patients [b:4.52 ± 2.24 (*P* = .04)].

**Conclusions:**

Group-specific behavioral barriers are important when improving self-care behaviors in patients with hypertension. Perceived control over self-care behaviors is more important in vulnerable patients, such as the elderly and women.

## Background

Hypertension is one of the most important preventable contributors to morbidity and mortality [1–3]. Following the 2013 World Health Organization (WHO) report, cardiovascular diseases (CVDs) cause approximately 17 million deaths yearly globally, and hypertension is responsible for at least 55% of these deaths [1]. It is estimated that there would be more than 500 million (about 60%) hypertensive patients around the world by the year 2025. Hypertension is considered to be a major modifiable risk factor for CVDs, resulting in more deaths than any other risk factor in the Asian regions [4]. The incidence of hypertension is associated with salt intake, stress level, and obesity [5, 6], and it is higher in low- and middle-income countries where patients have less awareness regarding the importance of hypertension control [6, 7]. According to the recent reports, about one-quarter of Iranian adults have hypertension (systolic blood pressure ≥ 140 or diastolic blood pressure ≥ 90), of which 56.6% are men, and 43.4% are women [1, 8].

The control of hypertension is one of the most important health goals worldwide. There is strong evidence that self-care management that involves the evaluation of any physical changes and deciding whether these changes need to be addressed has a significant effect on hypertension control [8–10]. Recent evidence-based guidelines for the management of hypertension among adults from the Joint National Committee-8 (JNC-8) contains eight recommendations for controlling hypertension through patient’s behavior:1.proper use of antihypertensive drugs; 2.home blood pressure control; 3.stress and anxiety control; 4.alcohol and smoking cessation; 5.physical activity; 6.weight loss; 7.Compliance; and 8.low-salt or DASH diet (Dietary Approaches to Stop Hypertension). However, changing individual patient behavior and lifestyle is a complex process [11–13]. Studies have shown that cognitive components of self-care behaviors are as important as the patient’sdemographic characteristics [14]. Therefore, studying self-care behaviors through cognitive behavioral models may reveal important information for designing an effective intervention to improve self-care behaviors [15].

The theory of planned behavior (TPB) links one’s beliefs and behavior. This theory, proposed by Icek Ajzen, has been widely applied to study a variety of behaviors in various fields, including advertising, public relations, and healthcare [16]. TPB states that intention toward attitude, subject norms, and perceived behavioral control, together shape an individual’s behavioral intentions and behaviors. It extends the theory of reasoned action by adding the concept of perceived behavioral control, defined as an individual’s perception of the ease or difficulty of performing the particular behavior [14].TPB has shown more utility in public health than other models, such as the Health Belief Model. It is also more applicable when the probability of success and actual control over the performance of a behavior is suboptimal [17].

We used TPB to determine important variables that affect self-care behaviors in the control of hypertension. We also estimate the prevalence of performing the acceptable self-care behavior and the score of the patient’s knowledge.

## Methods

This cross-sectional study was conducted between April 2016 and August 2017. Using a consecutive sampling method, five hundred fourteen patients with at least one-year history of diagnosed hypertension,underthe medication and without major consequences of hypertension, referred to the heart clinic of SinaUniversity Hospital for their regular check-up, were invited to participate in the study. One of researchers was staying in heart clinic and was inviting eligible patients to participate in the study. The invitation was continued until the sample size (500 cases) agreed to participate. The hospital’s reference population belongs primarily to low and middle socio-economic strata.

The data collection tool wasdesigned based the manual of constructing questionnaires based on the TPB [16, 18] and self-care behaviors in JNC-8 [19]. It had three parts. Part 1: 23 questions with four sections: A-attitude towards behavior (six questions), B-subjective norms (six questions), C-perceived behavioral control (six questions), and D-behavioral intention (five questions). TPB questions were scored on a seven-point Likert scale. Part 2: questions on different self-care behavior status mentioned in JNC-8:six questions about knowledge and five questions about the behavior of eight different groups (including proper use of antihypertensive drugs, home hypertension control, stress and anxiety control, alcohol and smoking cessation, physical activity, weight loss, compliance low-salt diet or DASH diet which was considered as self-care behaviors). Part 3: questions on general and demographical information of the study population, as well as questions on knowledge and awareness about different self-care behaviors. The content of the questionnaire was discussed during several rounds among members of the multidisciplinary research team. The reliability and validity of the questionnaire were tested twice on 20 patients, using test-retest methods with 2 weeks’ time gap. Understanding of questions was clarified using the think-aloud strategy. We asked patients what they had thought when they were answering a specific question. The final questionnaire’smean ICC was 0.93 (0.68–1.00). The final questionnaire was designed as a self-administered questionnaire, however, in the data gathering stage, to make sure all questions were understood well by respondersand and to get a high level of response rate,one of researchers (RA) accompanied with every single of responders,

### Statistical analysis

Descriptive statistics and measurement of the relationship between independent variables and self-care behaviors were performed using Stata14. Knowledge and behavior scores were calculated by summing questions in part 2 of the questionnaire. Then they were standardized in a way to shape two 10-point scale variables named Knowledge and Behavior. Patients who scored≥8 points were considered as having good knowledge and acceptable behavior.

**Fig. 1:**
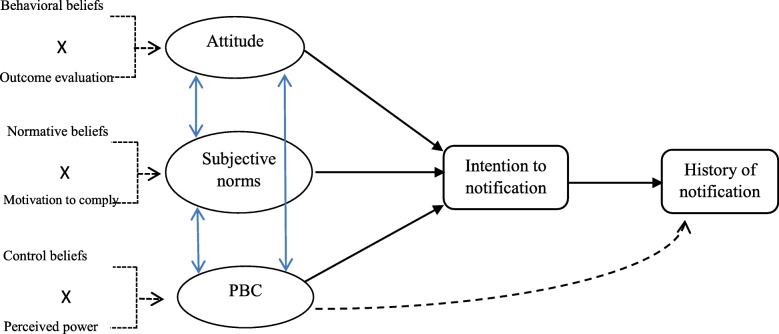
Theory of planned behavior (Azjen 1991). Note: Within the TPB, the determinants of behavior are intentions to engage in that behavior and perceived behavioral control (PBC) over that behavior. Intentions are determined by three variables. The first is attitudes, which are an individual’s overall evaluation of the behavior. The second is subjective norms, which consist of a person’s beliefs about whether significant others think he/she should engage in the behavior. The third measure, the extent to which the individual perceives that the behavior is under their control and is labeled PBC. (Azjen 1991; Reproduce permission from Elsevier has obtained. License Number: 4880610612547; Aug 02, 2020 [16])

**Fig. 2:**
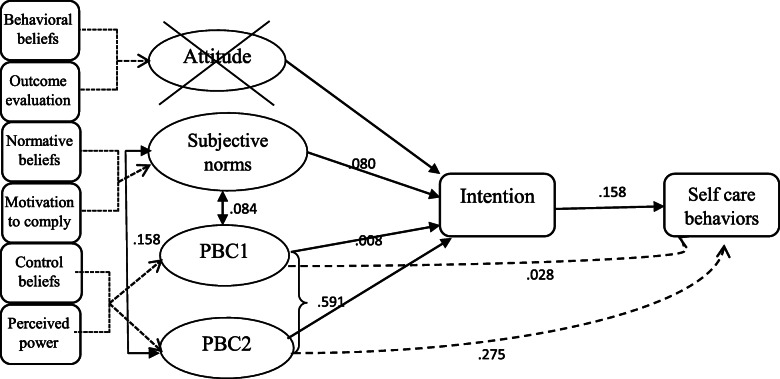
The final model of theory of planned behavior and the estimation of its parameters for self-care behaviors in patients with hypertension. *Attitude was removed because of low variance. **PBC1: Perceived behavioral control for proper use of antihypertensive drugs, home blood pressure control, stress and anxiety control, physical activity, weight loss, compliance, low-salt intake. ***PBC2: Perceived behavioral control alcohol and smoking cessation

Confirmatory Factor Analysis (CFA) was used to score exogenous factors (attitude, subjective norm, Perceived Behavioral Control). CFA yielded scores of participants’ attitudes, subjective norms, and perceived behavioral controls, using loading scores of Atitiute (ATT), Subjevtive Norms (SN), and Percived Bahvioural Control (PBC) indicators, respectively. TheStructural Equation Modeling (SEM) was used to develop and specify the TPB model, using AMOS software (Fig. 1). We used the Maximum Likelihood (ML) approach [20]. SEM analysis was performed using a two-stage structural equation model. RMSEA and CFI were the main fitting indices based on their acceptance values (RMSEA = 0.08, CFI close to 1). Behavior and intention were considered as endogenous variables, and attitude, subjective norms, and perceived behavioral control were considered as exogenous variables. The role of intention was analyzed as a mediator in the model. In the final model, the attitude factor was removed from the model analysis because it had a low variance (Mean 0.998; SD ± 0.008,range: 0–1), which made it unapplicable as a predictor in the analysis. In the PBC component, alcohol and smoking cessations were taken account in a separate group (as PBC2) due to the type of questions and statistical results that showed very different loading factor on the PBC component in the measurement model (Fig. 2). Additionally, only a subset of patients had a history of smoking and alcohol consumption, which was not the case for other PBC components. We first performed analyses for all patients together and then stratifyingby age and sex. Mean imputation was used for replacing the missing values in the response variables.

## Results

Five houndered fourteen patients were invited to compelete sample size of 500 patients. Fourteen patients rejected to participate in the study due to their health status or lack of interest (response rate 97.3%). The mean ± sd age of the participants was 62.6 ± 9.7 years (min = 42, max = 88). Half of the patients were under 62 years old. Younger patients had better knowledge regarding self-care behaviors (*p* = .01). The mean ± se of systolic and diastolic blood pressure at the time of study were 135.18 ± .42 and 110.75 ± .74. The mean change of the systolic blood pressure, was 36.82 ± .38. This change was higher in patients with acceptable behavior (mean ± se change 39.9 ± 1.5 for the acceptable behavior group compared to 36.9 ± .45 and 34.8 ± .82 for those with moderate behavior and unacceptable behavior, respectively, *p* = 0.012). For diastolic blood pressure the mean ± se of change was 24.78 ± .83. It was not statistically significant over behavior levels (30.05 ± 5.43; 23.99 ± .84; 25.63 ± 1.91 for acceptable, moderate and unacceptable level of behavior, respectively; p_value:0.13). For the knowledge, the mean ± se for systolic and diastolic blood pressure were not different (for systolic changes they were 37.56 ± .98; 36.51 ± .45; 37.27 ± 1.06 for acceptable, moderate and weak level of knowledge, respectively; p_value:0.53 and for diastolic changes they were 25.65 ± 1.81; 24.17 ± .94; 26.65 ± 3.16 for acceptable, moderate and weak level of knowledge, respectively; p_value:0.28). Finally, there was a relatively strong correlation between age and the duration of disease (r = .716, *p* < .001). Other baseline characteristics and their relationship with Knowledge and Behavior are in Table 1.

**Table 1:** Demographic and clinical characteristics of patients

Variables		N(%)	Knowledge^a^(mean ± se)	Behavior^a^(mean ± se)
Age	< 60	198(39.6)	6.82 ± .10	6.12 ± .09
	60–70	189 (37.8)	6.78 ± .10	6.25 ± .09
	> 70	113 (22.6)	6.34 ± .12	6.30 ± .12
			*P* = .013	*P* = .458
Sex	Female	217 (43.4)	6.70 ± .10	6.14 ± .07
	Male	283 (56.6)	6.69 ± .08	6.69 ± .08
			*P* = .961	*P* = .172
Education	Illiterate	85 (17.0)	6.19 ± .16	6.21 ± .14
	< High school diploma	142 (28.4)	6.67 ± .11	6.36 ± .10
	High school diploma	159 (31.8)	6.94 ± .11	6.13 ± .10
	Academic >Bachelor’s degree	114 (22.8)	6.76 ± .14	6.13 ± .13
			*P* = .002	*P* = .418
Duration of hypertension (months)	1–3 yrs.	188 (37.9)	6.76 ± .10	6.25 ± .10
	3–6 yrs.	149 (30.0)	6.85 ± .11	6.32 ± .10
	6–8 yrs.	58 (11.7)	6.61 ± .18	6.36 ± .14
	8 yrs. <	102 (20.5)	6.40 ± .15	6.03 ± .14
			*P* = .101	*P* = .435
Smoking	Yes	124 (24.7)	6.26 ± .14	6.26 ± .11
	No	376 (75.2)	6.84 ± .07	6.196 ± .06
			*P* = .001	*P* = .597
Alcohol use	Yes	76 (15.2)	6.76 ± .16	6.226 ± .06
	No		6.696 ± .07	6.15 ± .13
			*P* = .956	*P* = .690

^a^Knowledge and behavior were calculated by summing questions in the part 2 of the questionnaire. They then were standardized in a way to shaping two 10 scales variables named as Knowledge and Behavior

The SEM analysis results in all patients are presented in Fig. 2 and Table 2. According to the final SEM model, the perception of having control over quitting smoking and alcohol intake were associated with the patient’s intention and (self-care) behavior (b:1.283 ± .095 and b:1.59 ± .014,*p* < .001, standard estimates were .59 and .27, respectively). The intention had a significant relationship with behavior (b:.04 ± .03, *p* < .001, standard estimate = .15). Subgroup analysis based on age and gender is shown in Table 3.In all age groups, PBC2(perceived control over quitting smoking and alcohol intake) had a significant relationship with intention and behavior. It was stronger in patients under 60 years old (standard estimate = .68, *p* < .001 for intention and .33, *p* > .001 for behavior) in comparison to patients between 60 and 70 (standard estimate = .56, *p* < .001 for intention and .20, *p* = .02 for behavior) and patients older than 70 (standard estimate = .49, *p <* .001 for intention and .29, *p >* .001 for behavior). It was the same for female (standard estimate = .68, *p <* .001 for intention and .26, *p <* .001 for behavior) and male patients (standard estimate = .56, *p <* .001 for intention and .23, *p <* .001 for behavior). PBC1(having control over lifestyle behaviors other than smoking and alcohol use) was a significant determinant of intention just in female patients (standard estimate = .14, *p* = .04). Subjective norms were an important determinant of intention in younger patients (standard estimate = .13, *p =* .04), while it was not significant in patients 60–70 years old and those older than 70 (standard estimate = .06, *p* = .41 and standard estimate = .08, *p* = 0.45, respectively).

**Table 2:** Main estimates for Theory of Planned Behavior parameters

Parameter	Estimate (±SE)	p	Standard estimate
INT^a^ < −--SN^b^	1.36 (±.89)	.12	.08
INT < ---PBC1^c^	.07 (±.50)	.88	.00
INT < ---PBC2^d^	1.28 (±.09)	.00	.59
BEH^e^ < −--PBC1	.07 (±.14)	.61	.02
BEH < ---PBC2	.15 (±.01)	.00	.27
BEH < ---INT	.04 (±.03)	.00	.15

^a^:INT = Behavioral intention, ^b^:SN = Subjective norms, ^c^:PBC1 = Percived behavioral control for proper use of antihypertensive drugs, home blood pressure control, stress and anxiety control, physical activity, weight loss, compliance, low-salt intake, ^d^:PBC2 = Perceived behavioral control alcohol and smoking cessation, ^e^:Beh = Blood pressure control behavior

**Table 3:** Sub group analysis of estimated relationships in TPB model based on age and sex

	Parameter	Estimate (±SE)	p	Standard estimate
	INT^a^<−--SN^b^	4.52 (±2.24)	.04	.13
	INT < ---PBC1^c^	.28 (±.53)	.59	.03
Age < 60	INT < ---PBC2^d^	1.27 (±.11)	.00	.68
*n* = 198	BEH^e^<−--PBC1	.08 (±.17)	.62	.03
	BEH < ---PBC2	.01 (±.04)	.00	.33
	BEH < ---INT	.00 (±.02)	.06	.16
	INT^a^<−--SN^b^	.86 (±1.043)	.41	.06
	INT < ---PBC1^c^	.047 (±.41)	.91	.01
Age:60–70	INT < ---PBC2^d^	1.36 (±.18)	.00	.56
*n* = 189	BEH^e^<−--PBC1	.04 (±.11)	.73	.03
	BEH < ---PBC2	.12 (±.05)	.02	.20
	BEH < ---INT	.03 (±.02)	.08	.15
	INT^a^<−--SN^b^	1.7 (±2.25)	.45	.08
	INT < ---PBC1^c^	.76 (±1.93)	.69	.09
Age > 70	INT < ---PBC2^d^	1.37 (±.33)	<.00	.49
*n* = 113	BEH^e^<−--PBC1	.09 (±.26)	.71	.04
	BEH < ---PBC2	.20 (±.08)	.01	.29
	BEH < ---INT	.03 (±.02)	.15	.14
	INT^a^<−--SN^b^	1.98 (±1.67)	.23	.05
	INT < ---PBC1^c^	.88 (±.44)	.04	.14
Female	INT < ---PBC2^d^	1.13 (±.09)	.00	.67
*n* = 217	BEH^e^<−--PBC1	.14 (±.14)	.33	.08
	BEH < ---PBC2	.12 (±.04)	.00	.26
	BEH < ---INT	.064 (±.025)	.01	.22
	INT^a^<−--SN^b^	1.98 (±1.39)	.15	.12
	INT < ---PBC1c	8.93 (±14.99)	.53	.08
Male	INT < ---PBC2^d^	1.50 (±.18)	.00	.56
*n* = 283	BEH^e^<−--PBC1	.57 (±1.40)	.67	.02
	BEH < ---PBC2	.16 (±.05)	.00	.23
	BEH < ---INT	.04 (±.01)	.00	.18

^a^:INT = Behavioral intention, ^b^:SN = Subjective norms, ^c^:PBC1 = Percived behavioral control for proper use of antihypertensive drugs, home blood pressure control, stress and anxiety control, physical activity, weight loss, compliance, low-salt intake, ^d^:PBC2 = Perceived behavioral control alcohol and smoking cessation, ^e^:Beh = Blood pressure control behavior

## Discussion

Perceived behavioral control was the main determinant of self-care behavior in patients with hypertension in this study. Patients who believe that they have low control over their behaviors were less likely to have a strong intention to perform self-care behavior and consequently less likely to perform self-care behavior. Besides, our results show that the strength of relationships between model components and the behavior in different subgroups of age and sex were not the same. Female and older patients felt less control over their behavior. In elderly patients, the intention had a weaker relationship with the behavior, emphasizing the necessity to support this age group in order to improve their behavior. In female patients, the PBC components (other than smoking and alcohol intake) was also an effective determinant of both the intention and the behavior. The probable interpretation of this result is that these groups of patients might not have enough control over their lifestyle behaviors primarily due to cultural circumstances and expectations. Most of the female patients were housewives or homestay mothers who are responsible for meeting all family lifestyle needs [21]. We suggest that interventions that aim at empowering patients to make positive decisions in their lifestyle be considered for more assessment. More precisely, the empowerment of patients to control their behavior is indicatedas a probable effective intervention to improve self-care behavior. WHO defines empowerment as a process through which people gain greater control over decisions and actions affecting their health rather than being passive recipients of health care [15]. Patient empowerment is now one of the main policy interventions in health systems globally [22]. It uses shared decision-making to improve patient engagementand increase their confidence in their ability to exert control over their health and healthcare. According to Ellen Selman (2017), empowering patients in one setting needs changes throughout the health system. Social participation, good staff-patient communication, increasing the knowledge level of the family, patient-centred care, an organizational focus on patient experience rather than throughput, and appropriate access to palliative care are the most influential factors on empowerment of patients [23, 24]. Our results also showed that younger patients are more impressed by subjective norms, and we can conclude that having communication with other patients would be helpful for younger patients (since we measured subjective norms as “what other patients do in this case”). We also suggest establishing a social network to be considered as an intervention to promote self-care behaviors, specifically for younger patients.

Although our study result indicates that empowerment interventions might be effective for such patients, using universal intervention aiming to improve self-care behaviors most likely will not have the same efficacy for all patients subgroups. Any intervention to improve self-care behavior in patients with hypertension should be adjusted for patient-specific barriers to behavior change [25]. Due to the difference in awareness, understanding, and ability of patients regarding the performing self-care behavior, the multi-dimensional approach might increase the power of behavioral control [18]. Furthermore, in the elderly group of patients, the ability to communicate with healthcare professionals may be troublesome for them, which can be due to age-related physiological changes or language barriers [10]. The longer duration of the disease in eldery patients should also be considered in the interperetation of age related estimations. Evidence suggests that self-care management,especially in senior people, is a collective process undertaken within social networks and personal communities that requires the mobilization social resources [26, 27].

Using the SEM technique allowed us to estimate the inferences of the causal relations between attitude, perceived behavioral control, behavioral intention, and implementation intention. Concerning the perceived behavioral control, different types of self-carebehaviorin people with hypertensionhad different degrees of difficulty to be performed. In our study, smoking cessation and reducing alcohol intake were the most difficult behaviors, and increasing physical activity was the second most difficult challenge. It was found out that the perceived control over smoking and alcohol use was quite different from other behaviors. So we considered PBC for smoking and using alcohol separately (PBC2). It should be considered that compared to other behaviors (referred to as positive behaviors),smoking and alcohol consumption are being regarded as misbehaviors and are accompanied by some degree of a social stigma [28]. This difference can be due to the proportion of smokers and alcohol users in our study sample (which makes wider CIs around estimations), or the stigma around these behaviors (which makes them more susceptible to social desirability bias [29]). Other explanation around this result may be that the patients truly perceived less power over control these two behaviors in comparison to other behaviors. In sum, with regards to the high power of the perceived behavioral control and the high positive attitude towards self-care behaviors, interventions focusing on leading intention to behavior [30, 31] can be considered for more assessment.

### Study limitation

The study sample frame included patients who have not suffered major consequences of hypertension such as cardiovascular and renal complications. They were referred to the clinic for their regular cardiovascular check out. The hospital’s reference population for such services is likely to be the geographical area around the hospital that most of them belong to low and middle socio-economic status. However, we cannot say that our sample is a representative sample of a defined population, at least for selection bias due to referring to hospital and being free of the disease major consequences. In addition, the study methodology of data gathering had some extend of limitation. The measured behavior of notification in this study was past behavior. Although measuring past behavior is commonly used in behavioral studies, it makes results susceptible to some systematic (social desirability bias) and accidental measurement errors [29, 32]. Finally, because of sample size limitation, we could not run stratification for more than one variable simoultaniously. For example we were not able to decompose the modification effect of the age from the duration of disease.

## Conclusion

This study showed that the perception of having control over self-care behaviors in patients is an important determinant of performing self-care behavior. Therefore, it can be considered as an important barrierforplaning interventions which aim to enhance intention toward self-care behavior. It is more crucial in vulnerable patients, such as the elderly and women. We suggest interventions aimed to empower for patients with less control over their self-care behaviors and planning group-specific behavioral interventions to be considered for improving self-care behaviors in such patients.

## Data Availability

All data generated or analysed during this study are included in this published article [and its supplementary information files].

## Abbreviations

CVD: Cardiovascular Disease
JNC8: Joint national committee-8
DASH: Dietary Approaches to Stop Hypertension
TPB: Theory of Planned Behavior
WHO: World Health Organization
SEM: Structural Equation Model
ML: Maximum Likelihood
PBC: Perceived Behavioral Controls
ATT: Atitiute
SN: Subjevtive Norms
CFA: Confirmatory Factor Analysis

## References

[1] World Health Organization. A global brief on hypertension: silent killer, global public health crisis: World Health Day 2013: WHO; 2013. WHO_DCO_WHD_2013.2_eng.

[2] Adeloye D, Basquill C, Aderemi AV, Thompson JY, Obi FA (2015). An estimate of the prevalence of hypertension in Nigeria: a systematic review and meta-analysis. J Hypertens.

[3] Schmidt B-M, Durao S, Toews I, Bavuma CM, Hohlfeld A, Nury E, Meerpohl JJ, Kredo T. Screening strategies for hypertension. Cochrane Database Syst Rev. 2020;(Issue 5):Art. No.: CD013212. 10.1002/14651858.CD013212.pub2. 10.1002/14651858.CD013212.pub2 PMC7203601 32378196

[4] Soenarta AA, Buranakitjaroen P, Chia YC, Chen CH, Nailes J, Hoshide S, et al. An overview of hypertension and cardiac involvement in Asia: focus on heart failure. J Clin Hypertens. 2020. 10.1111/jch.13753 PMC8029815 31955506

[5] Namayandeh S, Sadr S, Rafiei M, Modares-Mosadegh M, Rajaefard M (2011). Hypertension in Iranian urban population, epidemiology, awareness, treatment and control. Iran J Public Health.

[6] Kearney PM, Whelton M, Reynolds K, Muntner P, Whelton PK, He J (2005). Global burden of hypertension: analysis of worldwide data. Lancet (London, England).

[7] Ibrahim MM, Damasceno A (2012). Hypertension in developing countries. Lancet.

[8] Mirzaei M, Moayedallaie S, Jabbari L, Mohammadi M (2016). Prevalence of Hypertension in Iran 1980–2012: A Systematic Review. J Tehran Heart Cent.

[9] Campbell NR, Niebylski ML (2014). Prevention and control of hypertension: developing a global agenda. Curr Opin Cardiol.

[10] Yang SO, Jeong GH, Kim SJ, Lee SH (2014). Correlates of self-care behaviors among low-income elderly women with hypertension in South Korea. J Obstet Gynecol Neonatal Nurs.

[11] Jones DW, Hall JE (2004). Seventh Report of the Joint National Committee on Prevention, Detection, Evaluation, and Treatment of High Blood Pressure and Evidence From New Hypertension Trials. Hypertension.

[12] James PA, Oparil S, Carter BL, Cushman WC, Dennison-Himmelfarb C, Handler J (2014). 2014 evidence-based guideline for the management of high blood pressure in adults: report from the panel members appointed to the eighth joint National Committee (JNC 8). Jama..

[13] Elhani S, Cleophas TJ, Atiqi R (2009). Lifestyle interventions in the management of hypertension: a survey based on the opinion of 105 practitioners. Neth Hear J.

[14] Douglas BM, Howard EP (2015). Predictors of self-management behaviors in older adults with hypertension. Adv Prev Med.

[15] World Health Organization (2013). Health 2020: a European policy framework and strategy for the 21st century: WHO. Regional Office for Europe.

[16] Ajzen I (1991). The theory of planned behavior. Organ Behav Hum Decis Process.

[17] Mayhew MJ, Hubbard SM, Finelli CJ, Harding TS, Carpenter DD (2009). Using structural equation modeling to validate the theory of planned behavior as a model for predicting student cheating. Health Psychol.

[18] Michie S, Abraham C, Whittington C, McAteer J, Gupta S (2009). Effective techniques in healthy eating and physical activity interventions: a meta-regression. Health psychology : official journal of the division of health psychology, American Psychological Association. Health Psychol.

[19] Reisin E, Harris RC, Rahman M (2014). Commentary on the 2014 BP guidelines from the panel appointed to the eighth joint National Committee (JNC 8). J Am Soc Nephrol.

[20] Bollen KA. Wiley series in probability and mathematical statistics. In: Applied probability and statistics section Structural equations with latent variables: John Wiley & Sons; 1989. 10.1002/9781118619179.

[21] Krubiner CB, Salmon M, Synowiec C, Lagomarsino G (2016). Investing in nursing and midwifery enterprise: empowering women and strengthening health systems--a landscaping study of innovations in low- and middle-income countries. Nurs Outlook.

[22] Bailo L, Guiddi P, Vergani L, Marton G, Pravettoni G (2019). The patient perspective: investigating patient empowerment enablers and barriers within the oncological care process. Ecancermedicalscience.

[23] Selman LE, Daveson BA, Smith M, Johnston B, Ryan K, Morrison RS (2017). How empowering is hospital care for older people with advanced disease? Barriers and facilitators from a cross-national ethnography in England, Ireland and the USA. Age Ageing.

[24] Hedayati B (2015). The effect of implementation of family-centered empowerment model on the self-esteem of the old people with hypertension. J Educ Health Promot.

[25] Liu S, Tanaka R, Barr S, Nolan RP (2020). Effects of self-guided e-counseling on health behaviors and blood pressure: results of a randomized trial. Patient Educ Couns.

[26] Bantum EO, Albright CL, White KK, Berenberg JL, Layi G, Ritter PL (2014). Surviving and thriving with cancer using a web-based health behavior change intervention: randomized controlled trial. J Med Internet Res.

[27] Vassilev I, Rogers A, Kennedy A, Koetsenruijter J (2014). The influence of social networks on self-management support: a metasynthesis. BMC Public Health.

[28] Evans-Polce RJ, Castaldelli-Maia JM, Schomerus G, Evans-Lacko SE (2015). The downside of tobacco control? Smoking and self-stigma: a systematic review. Soc Sci Med.

[29] Bou Malham P, Saucier G (2016). The conceptual link between social desirability and cultural normativity. Int J Psychol.

[30] Sheeran P (2002). Intention—behavior relations: a conceptual and empirical review. Eur Rev Soc Psychol.

[31] Francis J, Eccles MP, Johnston M, Walker A, Grimshaw JM, Foy R (2004). Constructing questionnaires based on the theory of planned behaviour: A manual for health services researchers. Centre for Health Services Research, University of Newcastle upon Tyne.

[32] Manning M (2009). The effects of subjective norms on behaviour in the theory of planned behaviour: a meta-analysis. Br J Soc Psychol.

